# Supplementary material for: Multimodal neuromarkers in schizophrenia via cognition-guided MRI fusion
Source: Nat Commun. 2018 Aug 2;9:3028. doi: 10.1038/s41467-018-05432-w (PMC6072778; doi:10.1038/s41467-018-05432-w)
Supplement: Supplementary file 1 — Supplementary Information [file 41467_2018_5432_MOESM1_ESM.pdf]

Supplementary Information

Multimodal neuromarkers in schizophrenia via cognition-guided MRI fusion

Sui, Qi, et.al.

Supplementary Table 1. Anatomical information of the identified joint FBIRN_IC$_{ref_composite}$

fMRI_fALFF Area	Brodmann Area	volume (cc)	random effects: Max (x, y, z)R/L
HC>SZ			
Parahippocampal Gyrus	19, 30	0.6/1.9	3.0 (-18, -56, -5)/3.9 (24, -52, 5)
Lingual Gyrus	17, 18, 19	4.7/2.4	3.3 (-21, -61, -2)/3.6 (27, -58, 3)
Fusiform Gyrus	19, 37	0.5/1.2	2.8 (-21, -62, -7)/2.5 (24, -56, -7)
Superior/ middle Temporal Gyrus	21,22,38	0.0/3.1	NA/2.7 (62, -3, -5)
SZ>HC			
Cingulate Gyrus	23, 24, 31	6.1/6.4	5.1 (0, -31, 26)/4.8 (3, -31, 29)
Posterior Cingulate	23, 29, 30, 31	3.9/2.7	3.9 (-3, -37, 24)/4.4 (3, -31, 24)
Precuneus	7, 19, 23, 31, 39	19.1/9.5	4.3 (-3, -62, 45)/3.5 (3, -59, 53)
Angular Gyrus	39	3.3/0.7	4.2 (-48, -65, 34)/2.9 (45, -68, 31)
Posterior/inferior Parietal Cortex	7,39,40	3.5/1.0	3.7 (-3, -64, 53)/2.7 (12, -67, 53)
Middle Temporal Gyrus	19, 21, 37, 39	2.2/2.9	3.6 (-48, -66, 28)/3.4 (48, -69, 26)
Superior Occipital Gyrus	19	0.6/0.6	3.1 (-39, -77, 31)/2.7 (45, -77, 29)
Middle/inferior Frontal Gyrus	10, 46	1.7/0.1	3.0 (-42, 47, 3)/2.1 (45, 49, -2)
Superior/inferior Temporal Gyrus	22, 37,39	0.3/0.4	2.7 (-48, -60, 28)/2.7 (50, -60, 20)
**sMRI_GM**	**Brodmann Area**	**volume (cc)**	**random effects: Max (x, y, z) R/L**
HC>SZ			
Caudate		3.6/3.4	5.1 (-9, 15, 8)/4.5 (12, 18, 7)
super/medial/inf Frontal Gyrus	6, 9-11, 32, 46, 47	2.2/2.3	4.2 (-24, 36, 26)/3.2 (3, 50, 0)
Thalamus		1.1/0.6	3.4 (-6, -14, 17)/2.9 (9, -14, 17)
Anterior Cingulate	10, 25, 32	0.8/1.1	2.9 (0, 39, 20)/3.2 (3, 47, 3)
Superior/middle/inferior Temporal Gyrus	20,22, 37,38,39,40	2.1/1.5	2.9 (-45, 17, -13)/3.1 (53, 14, -8)
Cingulate Gyrus	32	0.1/0.2	2.4 (-3, 36, 26)/2.7 (3, 36, 26)
Inferior Parietal Lobule	40	0.1/0.0	2.6 (-36, -36, 38)/NA
Parahippocampal Gyrus	30	0.4/0.0	2.5 (-24, -49, 5)/NA
Insula	13	1.0/0.4	2.5 (-42, 11, -3)/2.3 (39, 17, -1)
SZ>HC			
Superior/middle Frontal Gyrus	6, 9	2.4/1.5	5.7 (-33, 19, 32)/3.6 (36, 33, 23)
Superior/inferior Parietal Lobule	7, 39, 40	1.2/0.9	3.3 (-48, -38, 57)/3.0 (48, -35, 57)
Superior/middle Temporal Gyrus	37	0.5/0.1	2.8 (-48, -43, 8)/2.0 (59, -32, -6)
**DTI_FA WM tracts**	**vol(cc)**	**Percentage**	**Z score Max(x, y, z)R/L**
HC>SZ			
Forceps minor/ forceps major	27.5/32.4	48%/64%	4.8(23,45,25)/3.6(29,26,24)
Superior longitudinal fasciculus	47.4/49.6	51%/48%	4.6(30,32,27)/4(29,39,26)
Cingulum (cingulate gyrus)	14.2/19	76%/72%	4.4(24,44,26)/4.6(30,41,27)
Anterior thalamic radiation	35.4/38.9	73%/74%	4.1(21,34,30)/4.1(30,32,24)
Corticospinal tract	24.2/20.6	66%/58%	3.1(20,35,30)/3.1(34,29,29)
Inferior fronto-occipital fasciculus	30.9/21.5	60%/52%	2.9(16,16,21)/3(37,15,22)
Inferior longitudinal fasciculus	23.5/24	55%/51%	2.8(17,14,21)/2.8(37,14,21)

Supplementary Table 2. Anatomical information of the identified joint UNM_IC$_{ref_composite}$

**fMRI_fALFF**	Brodmann Area	volume (cc)	random effects: Max (x, y, z) **R/L**
HC>SZ			
Posterior Parietal Cortex	40	4.4/4.5	5.1 (-56, -28, 29)/4.0 (62, -31, 26)
Superior/Middle/Inferior Temporal Gyrus	21, 22, 39, 41, 42	4.0/10.8	3.3 (-56, -24, -4)/4.8 (56, -43, 8)
Postcentral Gyrus	1, 2, 3, 40, 43	5.6/8.0	3.9 (-59, -25, 34)/4.2 (59, -19, 34)
Insula	13	2.6/1.3	2.9 (-42, -2, 8)/4.2 (50, -31, 21)
Posterior Cingulate	29, 30, 31	1.2/1.5	3.5 (-18, -63, 14)/2.8 (3, -46, 8)
Parahippocampal Gyrus	27, 30	0.0/0.7	NA/2.8 (9, -38, 2)
Lingual Gyrus	17, 18	0.1/0.5	2.3 (-12, -81, 4)/2.6 (15, -78, 4)
Thalamus		0.0/0.1	NA/2.2 (12, -32, 4)
SZ>HC			
Precuneus	7, 19, 31	11.4/14.7	4.7 (0, -50, 49)/5.0 (3, -56, 47)
Angular Gyrus	39	0.0/0.3	NA/4.4 (36, -71, 31)
Superior/Middle/Inferior Frontal Gyrus	6, 8, 9, 10, 44, 45, 46	0.9/8.1	2.6 (-30, 42, 34)/4.0 (53, 19, 27)
Paracentral Lobule	5, 31	2.0/0.8	3.7 (0, -41, 52)/3.6 (3, -41, 49)
Superior/Middle Occipital Gyrus	19,39	1.7/1.3	3.0 (-30, -84, 12)/3.3 (30, -78, 20)
Superior/Inferior Parietal Lobule	7, 40	0.8/2.7	2.5 (-24, -68, 45)/3.6 (12, -61, 56)
**sMRI_GM**	Brodmann Area	volume (cc)	random effects: Max (x, y, z) **R/L**
HC>SZ			
Caudate		3.5/3.0	4.9 (-9, 12, 10)/5.1 (9, 15, 10)
Thalamus		2.1/2.2	4.0 (-6, -17, 15)/4.0 (6, -14, 15)
Superior/ Middle/ Inferior Temporal Gyrus	21, 22, 37, 38, 39, 41, 42	5.0/5.4	4.0 (-53, 11, -6)/3.7 (53, 14, -3)
Middle/Inferior Frontal Gyrus	8, 9, 13, 44, 45, 46, 47	3.7/2.6	3.6 (-39, 11, -11)/4.0 (45, 14, -8)
Anterior Cingulate	24, 25	1.0/0.8	3.8 (-3, 11, -3)/3.5 (3, 11, -3)
Angular Gyrus		0.5/0.0	3.2 (-36, -54, 33)/NA
Parahippocampal Gyrus	19, 30	1.1/0.1	3.1 (-27, -50, -3)/2.1 (24, -38, 5)
Insula	13	0.6/1.0	2.9 (-42, 8, -5)/2.6 (45, 3, 0)
Inferior Parietal Lobule	40	0.1/0.9	2.1 (-42, -53, 39)/2.9 (50, -25, 26)
Middle Occipital Gyrus	19	0.0/0.3	NA/2.8 (39, -69, 17)
Precuneus	31	0.0/0.4	NA/2.8 (18, -66, 20)
Lingual Gyrus	18, 19	0.3/0.4	2.6 (-21, -49, 2)/2.6 (21, -70, -2)
Posterior Cingulate	23, 31	0.1/0.3	2.4 (-3, -28, 24)/2.3 (18, -63, 17)
Fusiform Gyrus	37	0.0/0.1	NA/2.1 (45, -50, -13)
SZ>HC			
Superior/Middle Frontal Gyrus	6, 8, 9, 10	1.8/2.2	3.3 (-27, 14, 44)/4.3 (39, 10, 33)
Inferior Parietal Lobule	40	0.3/0.5	2.6 (-30, -47, 47)/3.9 (48, -46, 22)
Superior/Middle Temporal Gyrus	13, 20, 22	0.4/2.4	2.6 (-48, -41, 0)/3.5 (50, -43, 8)
**dMRI_FA WM tracts**	**vol(cc)**	**Percentage**	**Z score Max(x, y, z)R/L**
HC>SZ			
Anterior thalamic radiation	31.2/34.8	65%/66%	5.6(26,35,23)/6(29,34,24)

Superior longitudinal fasciculus	30.5/37.9	33%/37%	3.7(30,32,27)/3(34,24,32)
Forceps minor/forceps major	29.9/20	53%/40%	3.2(32,51,19)/3.3(17,18,23)
Inferior fronto-occipital fasciculus	24.2/15.7	47%/38%	3.2(16,18,23)/3(40,26,18)
Inferior longitudinal fasciculus	16.2/19.1	38%/41%	2.5(15,19,22)/3(37,18,24)

Supplementary note 1: Permutation test

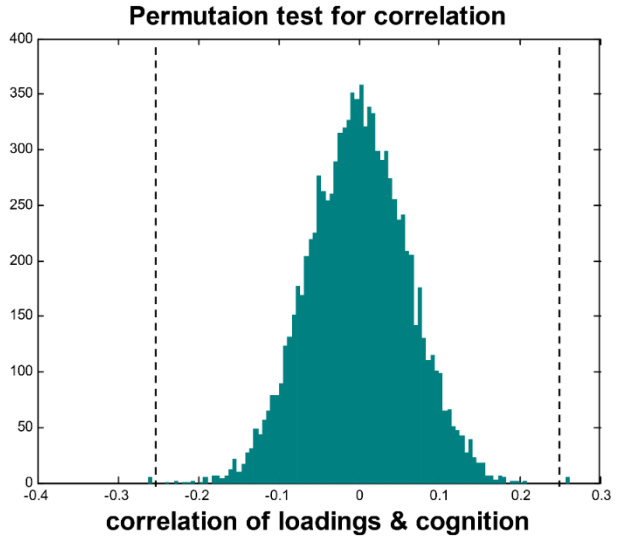

Supplementary Figure 1. Permutation test for the correlation analysis between FA_IC$_{ref}$ and cognitive scores (10000 times)**.** The black dotted line indicates $\pm$0.262.

We also performed standard permutation test for the correlations listed in the Results. We do this by randomly shuffling *Y* (cognitive scores) across participants and re-running the correlation analysis (between *X* [loadings of IC$_{ref}$] and *Y*) 10000 times in order to obtain an empirical null distribution. We also record the number of times a correlation coefficient between *X* and *Y* exceeds the obtained sample correlation (r = 0.262, here we take the FA component as an example). Significance cutoffs were determined using the above permutation test (10000 permutations). As shown Fig. 1b, the observed correlation between FA_IC$_{ref}$ and cognitive scores obtained on the original data was 0.262, while the sampling distribution of r under randomization is symmetric around 0.0 (Supplementary Figure 1), and 20 of the 10000 randomizations exceeded $\pm$0.262. This analysis quantifies the probability p = 0.002 of obtaining a particular r = 0.262 between loadings of FA_IC$_{ref}$ and composite cognitive scores by chance.

Based on the above permutation procedure, we tested all the correlations for both FBIRN and UNM. FBIRN: $p_{permutation}$ = 1.3×10^{-4}, 0.002, 1.0×10^{-4} for sMRI, dMRI and fMRI, respectively (Fig. 1b). UNM: $p_{permutation}$ = 0.02, 0.01, 0.001 for sMRI, dMRI and fMRI, respectively (Fig. 2b).

Supplementary note 2: Cross-cohort spatial correlation

We calculated the spatial correlation of the identified target component between two cohorts with only voxels masked at |Z| > T (threshold). First, the spatial maps were transformed into Z scores and masked at |Z| > 2. Then we obtained two masks from FBIRN (mask_FBIRN) and UNM (mask_UNM) respectively, which were used to perform the voxel selection. Only voxels that fell in the union of the masks (mask_FBIRN ∪ mask_UNM) were used to calculate the cross-cohort correlation. Thus total number of voxels in calculating the spatial correlation is greatly reduced. Here, take GM component, as an example, from n = 153594 (whole brain voxels) to m = 1936 (T = 2, used in our paper). Spatial correlation was finally performed on these commonly identified voxels (m = 1936) between two cohorts.

We further compared the impact of using different T thresholds on cross-cohort spatial correlations. As listed in Supplementary Table 3, all cross-cohort correlations r are significant (FDR corrected) regardless of different $\boldsymbol{T}$ thresholds, with $p < 1.0e\text{-}5$ in all cases.

Supplementary Table 3 Spatial correlation derived from different thresholded *T* values

Threshold	GM		FA		fALFF	
$T = 1$	$r = 0.38^*$	$m = 8553$	$r = 0.42^*$	$m = 7738$	$r = 0.22^*$	$m = 16420$
$T = 2$	$r = 0.51^*$	$m = 1936$	$r = 0.59^*$	$m = 2720$	$r = 0.39^*$	$m = 3692$
$T = 3$	$r = 0.65^*$	$m = 405$	$r = 0.67^*$	$m = 845$	$r = 0.45^*$	$m = 732$

Supplementary Table 4. CMINDS and PANSS associations with joint FBIRN_IC$_{ref_composite}$

IC_1	fMRI		dMRI		sMRI	
Corr with CMINDS	*r*	*p*	*r*	*p*	*r*	*p*
Composite	0.430	3.5E-13*	0.262	1.8E-05*	0.486	6.5E-17*
Speed of processing	0.287	1.8E-06*	0.236	1.0E-04*	0.414	1.7E-12*
Attention/vigilance	0.333	3.3E-08*	0.269	9.4E-06*	0.441	6.4E-14*
Working memory	0.341	1.0E-08*	0.240	7.2E-05*	0.402	8.1E-12*
Verbal learning	0.361	1.1E-09*	0.232	1.3E-04*	0.427	2.7E-13*
Visual learning	0.316	1.5E-07*	0.166	0.007	0.354	3.1E-09*
Reasoning/problem solving	0.185	0.002*			0.194	0.002
Correlation with PANSS-N			-0.162	0.050	-0.285	5.3E-04*

In addition, we also performed a permutation test to calculate the significance for the cross-cohort spatial correlation. We do this by randomly shuffling Y (UNM_IC$_{ref}$) across voxels and re-running the correlation analyses (between X [FBIRN_IC$_{ref}$] and Y) 10000 times in order to obtain an empirical null distribution. We then record the number of times the correlation exceeds the obtained sample

correlation. Take GM component for example (Fig. 3), the observed correlation between FBIRN_IC$_{ref}$ and UNM_IC$_{ref}$ was 0.51, while 8 of the 10000 permutations obtained correlations falling out the range of [-0.51, 0.51], thus the probability of $p = 8.0\times 10^{-4}$ was estimated for cross-cohort correlation of r = 0.51 between GM maps by chance.

Supplementary Table 5. Domain-specific IC$_{ref}$: correlation with the referred cognitive domain scores (*r*) and the group difference between HC and SZ (*p* of two sample t-test)

	fMRI		dMRI		sMRI	
**Corr with CMINDS domains**	***r***	***p***	***r***	***p***	***r***	***p***
**(FBIRN_IC$_{ref_attention}$)** Attention/vigilance	0.278	4.9e-06*	0.262	1.5e-05*	0.369	6.4e-10*
**(FBIRN_IC$_{ref_memory}$)** Working memory	0.296	7.8e-07*	0.241	6.7e-05*	0.301	5.0e-07*
**(FBIRN_IC$_{ref_learning}$)** Verbal learning	0.301	5.2e-07*	0.233	1.2e-04*	0.320	8.3e-08*

Supplementary Table 6. Partial correlation analysis of FBIRN results after controlling diagnosis (group label)

**Modality**	**GM_IC$_{ref}$**		**FA_IC$_{ref}$**		**fALFF_IC$_{ref}$**	
**Partial correlations**	***r***	***p***	***r***	***p***	***r***	***p***
Composite	0.241	9.0e-05*	0.118	0.05	0.275	6.9e-06*
Attention/vigilance	0.137	0.026	0.128	0.037	0.223	2.8e-04*
Working memory	0.291	1.3e-06*	0.188	0.002*	0.265	1.1e-05*
Verbal learning	0.151	0.014	0.156	0.001*	0.139	0.023

In order to quantify the robustness of the cognition-brain correlation across groups, we performed partial correlation to minimize the group effect. As shown in Supplementary Table 6, in any case of partial correlation, the cognition-imaging correlations remain significant (FDR corrected) after controlling for diagnosis in all four domains.

Supplementary Table 7. Correlation between CMINDS cognitive scores and the mean of the extracted ROI neuromarkers from Fig. 7a

**Extracted neuromarker ROI**	**GM_SN**		**FA_CC**		**fALFF_PFC**	
**CMINDS cognitive domain**	***r***	***p***	***r***	***p***	***r***	***p***
Composite	0.415	2.8e-12*	0.337	2.4e-08*	0.323	1.0e-07*
Speed of processing	0.432	1.5e-13*	0.375	2.3e-10*	0.226	2.0e-04*
Attention/vigilance	0.275	6.1e-06*	0.235	1.2e-04*	0.206	7.8e-04
Working memory	0.351	3.5e-09*	0.309	2.6e-07*	0.285	2.1e-06*
Verbal learning	0.362	1.1e-09*	0.249	3.9e-05*	0.227	1.8e-04*
Visual learning	0.414	2.1e-12*	0.358	2.0e-09*	0.290	1.6e-06*
Reasoning/problem solving	0.271	7.3e-06*	0.276	4.7e-06*	0.221	2.7e-04*

Supplementary note 3: Subgroup prediction

Moreover, we also performed the prediction analysis within each group (HC or SZ) based on the 4 neuromarker signatures, i.e., using the group model trained by FBIRN to predict UNM+COBRE. The generalization in either cases works well as shown in Supplementary Figure 2 below.

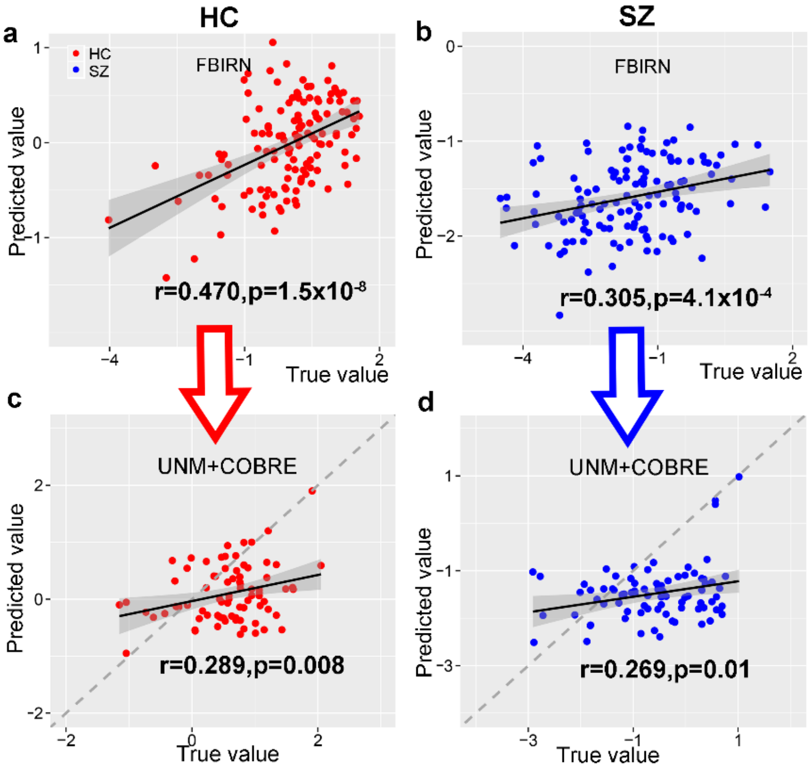

Supplementary Figure 2. **(a)** Prediction for HC group in FBIRN cohort. **(b)** Prediction for SZ in FBIRN cohort. (**c**) Generalization of model (a) to UNM+COBRE data for HC. (**d**) Generalization of model (b) to UNM+COBRE data for SZ. Both result in significant correlations.

Supplementary note 4: Medication information

Supplementary Table 8. Medication information of schizophrenia patients for FBIRN

	**SZ**
Unknown Medication History, (n) %	1 (0.6)
Medication data below are for subjects with medication history reported	n=146
No Medication taken, (n) %	0 (0)
Not on Psychotropic Medications, (n) %	0 (0)
On more than one Psychotropic Medications, (n) %	128 (87)
Antipsychotic (Any), (n) %	146 (99.8)
A. First Generation	15 (10.2)
B. Second Generation	131 (89.7)
Mood Stabilizer (Any), (n) %	30 (20.5)
A. Lithium	4 (13.3)
B. Anticonvulsants	26 (86.7)
Antidepressant (Any), (n) %	52 (34.4)
A. SSRIs/SNRIs	41 (78.8)
B. NDRI	5 (9.6)
C. MAO Inhibitors	0
D. Miscellaneous	6 (11.5)

Not surprisingly, most of the patients enrolled in our current study were taking antipsychotic medications. We performed correlation analysis between cognitive domain scores and medication dosages. A standardized total dose of drug dose, i.e., Chlorpromazine equivalent doses[1], were used to estimate medication dose. Supplementary Table 9 list the *p* values for correlations with all cognitive domains. It is clear that there is very little association between medication dose and cognitive scores in our current data.

Supplementary Table 9. Correlation analysis between medication dosages and cognitive scores

Cognitive domains	Composite	Speed of Processing	Attention	Working Memory	Verbal Learning	Visual Learning	Reasoning
p value	0.571	0.439	0.768	0.096	0.398	0.772	0.442
r	0.054	0.075	0.029	0.121	0.083	-0.028	0.074

Correlations between medication dosages and multimodal imaging features (voxel-wise) were calculated. No imaging voxels showed a significant correlation with medication dose, again suggesting that medication dose have little or at best very subtle effects on the brain imaging. These results support our claim that the identified replicable multimodal covarying patterns are associated with cognition

but not medication exposure.

Supplementary note 5: Parameter tuning of MCCAR+jICA

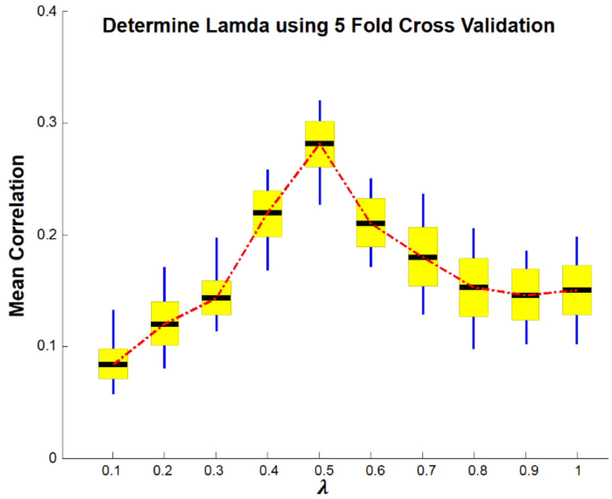

Supplementary Figure 3. Correlation of the identified components and CMINDS composite scores across multiple cross-validations. When λ is 0.5, the mean correlation (250 times) between estimated target IC and composite cognitive scores of all modalities reaches its maximum value. The black line, yellow patch and blue line represent mean, standard error of the mean (SEM) and the standard deviation (SD) of correlations between target IC and composite scores.

When determining the value of λ, we performed a five-fold cross validation on these 294 subjects for 50 iterations. 4/5 of the data was trained by MCCAR+jICA to be decomposed into A_{train} and S_{train} i.e., $X_{train} = A_{train} \times S_{train}$, where S_{train} is further used in the remaining 1/5 of testing data to obtain A_{test} ($A_{test} = X_{test} \times pinv(S_{train})$). Then we tested the correlation between the reference and the target component of A_{test} (with the same IC order of the target component derived from A_{train}) for 5×50 = 250 times on each modality. As shown in Supplementary Figure 3, the mean and standard derivation of correlations of all iterations for the three modalities were calculated and λ was set to the value at which the correlation between target IC and the reference reaches its maximum value (λ = 0.5 for the FBIRN data). For UNM data, we adopted the same strategy to independently determine the value of λ.

Supplementary Table 10. Demographics and the CMINDS scores for FBIRN subjects

Measure		HC	SZ	*p*	*r*
**Number**		147	147		
**Age**		37.4±11.1	39.5±11.8	0.117	-0.303
**Gender**		44F/103M	35F/112M	0.238	-0.139
**CMINDS**	Composite	-0.017±1.0	-1.590±1.2	1.7E-24	1
	Speed of processing	-0.010±1.0	-1.356±1.1	2.3E-21	0.729
	Attention/vigilance	0.002±1.0	-1.435±1.4	2.7E-18	0.770
	Working memory	0.010±1.0	-1.152±1.1	1.9E-17	0.731
	Verbal learning	0.024±1.0	-1.373±1.2	1.1E-21	0.785
	Visual learning	-0.017±1.0	-1.051±1.1	1.5E-13	0.830
	Reasoning/problem solving	-0.034±1.0	-0.803±1.2	6.6E-08	0.663

p denotes the significance value of two-sample t-tests performed between control subjects and schizophrenia patients. *r* is the Pearson correlation between CMINDS composite and the other measures. HC, healthy control subjects; F, female; M, male; CMINDS, the Computerized Multiphasic Interactive Neuro-cognitive System.

Supplementary Table 11. Demographic and site information of FBIRN data.

Site	Scanner	N(294)	SZ(147)	SZ Sex	SZ Age	HC(147)	HC Sex	HC Age
	All 3T	#subj	#subj	M/F	mean(std)	#subj	M/F	mean(std)
1-Duke	GE	36	16	13/3	35.0(10.6)	20	15/5	36.1(10.1)
2-Iowa	Siemens	21	12	11/1	45.3(10.8)	9	6/3	37.7(9.0)
3-UCI	Siemens	56	27	22/5	44.2(12.1)	29	21/8	42.4(13.1)
4-UCLA	Siemens	56	28	24/4	37.4(12.6)	28	22/6	35.9(11.3)
5-UCSF	Siemens	22	13	9/4	37.1(9.5)	9	6/3	39.3(9.3)
6-UMN	Siemens	54	27	15/12	36.7(10.9)	27	19/13	34.1(10.5)
7-UNM	Siemens	49	24	18/6	41.5(11.8)	25	19/6	37.2(9.9)

Supplementary Table 12. Demographics and the MCCB scores of UNM subjects

Measure		HC	SZ	p	r
**MCCB**	Composite	50.4±10.6	30.5±16.1	2.5E-08	1
	Speed of processing	52.1±9.2	34.5±14.4	2.2E-08	0.912
	Attention/vigilance	49.0±10.3	35.7±15.1	2.8E-05	0.864
	Working memory	46.9±11.4	35.8±14.8	3.8E-04	0.839
	Verbal learning	47.9±9.3	38.2±9.1	1.0E-05	0.810
	Visual learning	49.2±9.1	36.8±12.7	5.6E-06	0.787
	Reasoning/problem solving	48.8±9.3	36.8±12.7	10.0E-06	0.787
	Social cognition	54.8±9.8	45.8±11.4	0.5E-04	0.614

p denotes the significance value of two-sample t test performed between HC subjects and SZ patients

for all measures. *r* is the correlation value between MCCB composite and other measures. HC, healthy control subjects; MCCB, MATRICS Consensus Cognitive Battery.

All subjects of UNM were screened and excluded if they had a diagnosis of central neurological disorder or active substance use disorder (6-month minimum before enrollment, except for nicotine). In addition, HC subjects were excluded if they had first-degree relatives with any psychotic disorder. Patients met criteria for SZ defined by the DSM-IVTR based on the Structured Clinical Interview for DSM-IV Axis I Disorders, Patient Edition interview. All patients were clinically stable on the same antipsychotic medications > 4 weeks before the scan. Clinical assessment was performed within 1 week of scanning using the Positive and Negative Syndrome Scale (PANSS). Informed consent was obtained from all subjects according to institutional guidelines required by the Institutional Review Board. Subjects were paid for their participation.

Supplementary Table 13. Demographics and the MCCB scores of COBRE subjects

Measure		HC	SZ	*p*	*r*
**Number**		42	46		
**Age**		40.0±11.8	39.3±13.2	0.375	0.118
**Gender**		10F/32M	11F/35M	0.991	-0.019
**MCCB**	Composite	50.8±8.7	31.3±14.6	1.7E-10	1
	Speed of processing	53.6±9.0	33.3±11.8	4.0E-14	0.865
	Attention/vigilance	50.2±10.0	36.3±13.5	5.3E-07	0.852
	Working memory	50.3±9.8	39.6±13.6	4.9E-05	0.820
	Verbal learning	45.4±8.4	37.6±8.4	4.4E-05	0.722
	Visual learning	46.4±10.2	36.6±12.4	1.3E-04	0.719
	Reasoning/problem solving	57.2±7.3	44.0±11.9	4.0E-08	0.656
	Social cognition	51.5±10.6	42.3±12.5	3.0E-04	0.598

COBRE data：42 patients with schizophrenia and 46 age and gender matched healthy controls were included in the data set released from the Center for Biomedical Research Excellence (COBRE), University of New Mexico. All of the control participants were free of the DSM-IV diagnoses of schizophrenia and other mental disorders. None of all participants had neurological diseases, a history of any substance dependence, or a history of clinically significant head trauma. Informed consent was obtained from all subjects according to institutional guidelines required by the Institutional Review Board. Subjects were paid for their participation. The COBRE cohort also includes the MCCB cognitive battery.

Supplementary note 6: Head motion control

We remove outlier subjects who have framewise displacements (FD) exceeding 1.0 mm, as well

as head motion exceeding 2.0 mm of maximal translation (in any direction of x, y or z) or 1.0^{o} of maximal rotation throughout the course of scanning. We also despiked the fMRI data, and regressed out six head motion parameters, white matter, and cerebrospinal fluid. Results indicate all FDs (mean framewise displacements, mean of root of mean square frame-to-frame head motions assuming 50 mm head radius[2]) for all subjects were <0.3 mm at every time point. Note also there is no significant difference between patients and controls on mean FDs, namely,

UNM, HC: mean=0.22±0.12mm, SZ: 0.21±0.11 mm, two sample t-test: p = 0.77

FBIRN, HC: mean=0.25±0.18mm, SZ: 0.27±0.21mm, two sample t-test: p = 0.65

We also performed correlation analysis between cognitive scores and mean FDs for both FBIRN and UNM cohort, as displayed in Supplementary Table 14 and Table 15, none of these tests was significant.

Supplementary Table 14. *p* values for the correlations between mean FD and cognition for FBIRN

Composite	Speed of Processing	Attention	Working Memory	Verbal Learning	Visual Learning	Reasoning
0.650	0.602	0.450	0.365	0.782	0.685	0.562

Supplementary Table 15. *p* values for the correlations between mean FD and cognition for UNM

Composite	Speed of Processing	Attention	Working Memory	Verbal Learning	Visual Learning	Reasoning
0.139	0.166	0.096	0.112	0.153	0.371	0.637

We also performed partial correlation analysis for IC_{ref} and cognitive scores by regressing out mean FD, as shown in Supplementary Table 16 and Table 17, since partial correlation has been proposed as an alternative approach for removing spurious shared variance in correlation analysis[3]. It is clear that the correlations between components and cognitive scores are still significant after regressing out FD.

Supplementary Table 16. Partial correlation after regressing out mean FD for FBIRN results

**Modality**	**GM_IC$_{ref}$**		**FA_IC$_{ref}$**		**fALFF_IC$_{ref}$**	
**Partial correlations**	***r***	***p***	***r***	***p***	***r***	***p***
Composite	0.431	3.5e-11*	0.223	0.028	0.363	1.6e-07*
Attention/vigilance	0.318	1.4e-05*	0.233	0.001*	0.202	0.001*
Working memory	0.290	1.5e-04*	0.183	0.002*	0.232	0.0013*
Verbal learning	0.285	2.2e-04*	0.211	0.005	0.259	1.7e-03*

Supplementary Table 17. Partial correlation after regressing out mean FD for UNM results

Modality	GM_IC_{ref}		FA_IC_{ref}		$fALFF\_IC_{ref}$	
**Partial correlations**	***r***	***p***	***r***	***p***	***r***	***p***
composite	0.276	0.014	0.300	0.007*	0.304	0.006*

In FBIRN cohort, as for correlations between imaging features and mean FD, there is no imaging voxels showing a significant correlation with mean FD after FDR multiple comparison correction ($p_{uncorrected}$ < 1.0e-04) for fALFF, FA and GM. And the correlations between mean FD and fALFF_PFC and fALFF_pDMN as shown in Fig. 7a, are not significant either (p = 0.78 and p = 0.56). In UNM cohort, no imaging voxels showed a significant correlation with mean FD either for any of the 3 modalities after FDR correction ($p_{uncorrected}$ < 0.001). Considering there is no group difference in head motion, and no significant correlations between mean FD and cognitive scores, and partial correlations between IC_{ref} and cognitive scores are still significant after regressing out mean FD, we believe that micro-motion is not a major factor affecting the current results.

To test the specificity of the identified brain patterns to cognition but not motion, we also performed supervised fusion analysis using mean FD as reference. Results are shown in Supplementary Figure 4, the FD associated patterns are mainly artifacts in each modality, such as white matter in fALFF and GM, CSF in GM and FA.

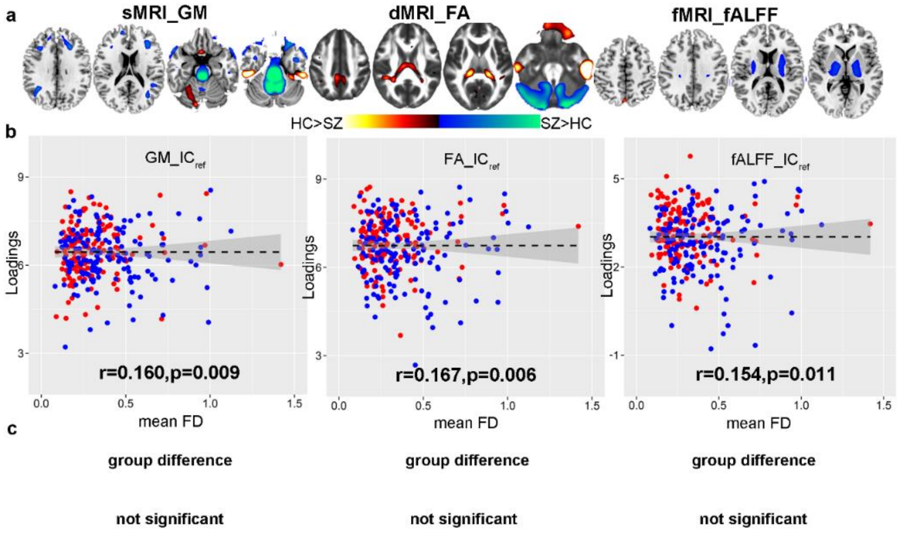

Supplementary Figure 4. The identified joint components that are significantly correlated with mean FD. (**a**) The spatial maps. (**b**) Correlations between loadings of component and mean FD (HC: the red dots, SZ: the blue dots). (**c**) There is no group difference for the loadings of components. The gray regions in (**b**) indicate a 95% confidence interval.

Supplementary note 7: Introduction of cognitive measurements

FBIRN: Cognitive measures were obtained from testing with the Computerized Multiphasic Interactive Neuro-cognitive System (CMINDS)[4]. Neurocognitive domain z-scores were calculated

from computerized neuropsychological tests, which are similar to those in the MATRICS Consensus Cognitive Battery (MCCB) system. The CMINDS includes computerized neuropsychological tasks that are structurally and functionally similar to standard paper-and-pencil neuropsychological tasks and allows for immediate electronic raw data capture and automated scoring of test results.

The CMINDS-based[4] cognitive domains, based on comparable tests to those assessed by the MCCB, were as follows: (1) *Speed of Processing*. This domain score was based on the mean of (a) the log-transformed, negated (worse performance is lower) elapsed time (in seconds) during *Trails A*, (b) the number of correct in set responses in 60 seconds on trial 1 of the *Category Fluency Test –Animals*, and (c) the number of correct responses during the *Symbol Digit Association Test* z-scores; (2) *Attention/Vigilance*. This domain score was based on the *d*-prime across blocks A–C of the *Continuous Performance Test z* -scores; (3) *Working Memory.* This domain score was based on the mean of (a) the sum of the number of correct on the *Visual Spatial Sequencing Test* – Forward and Backward condition, and (b) the total correct on the *Letter Number Span z* -scores; (4) *Verbal Learning.* This domain score was based on the total number of correctly recalled target words for all three trials on the *Semantic Verbal Learning Test* z-scores; (5) *Visual Learning.* This domain score was based on the square-transformed total of the *Visual Figure Learning Test z*-scores, and (6) *Reasoning/Problem Solving*. This domain score was based on the square transformed *Maze Solving Test* total score *z*-scores. Finally, the CMINDS composite score was defined as the mean of all six normalized domain scores."

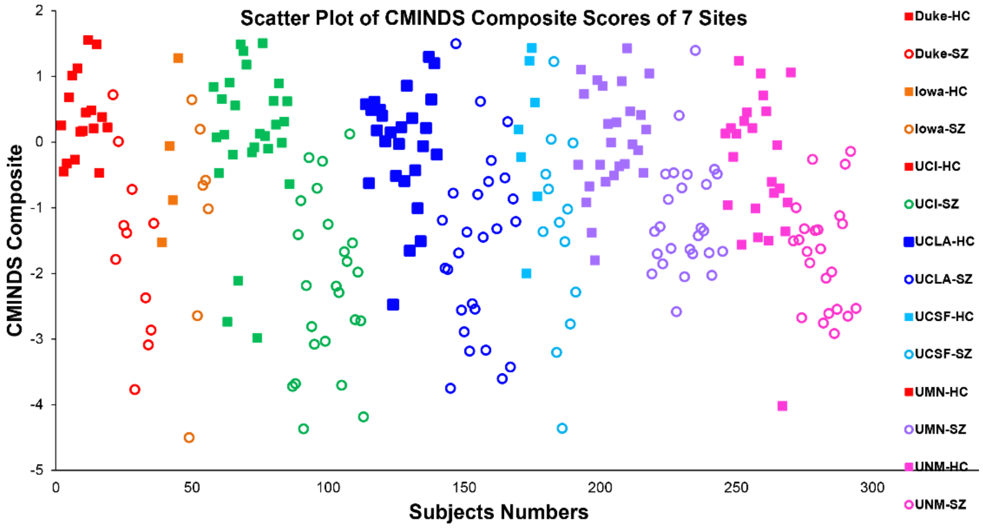

Supplementary Figure 5. Scatter plot of CMINDS composite scores of seven sites. The solid square and hollow circles represent HCs and SZs respectively.

UNM and COBRE: For the validated data cohort from UNM and COBRE, composite cognitive scores are measured by the MATRICS Consensus Cognitive Battery (**MCCB**) system, which was also launched by NIMH, and contains one more domain (social cognition) than CMINDS. As reported earlier[4], CMINDS is very similar to MATRICS on measuring cognitive deficits in SZ. The differences

in details between CMINDS and MCCB tasks have been previously cited[4]. The CMINDS scores of FBIRN III are listed in Supplementary Table 10. We also plot the CMINDS composite score of subjects from different sites, as seen in Supplementary Figure 5. It is apparent that the cognitive performance of HCs is better than SZs in all 7 sites.

Supplementary references

1. Woods SW. Chlorpromazine equivalent doses for the newer atypical antipsychotics. *The Journal of clinical psychiatry* **64**, 663-667 (2003).
2. Allen EA*, et al.* A baseline for the multivariate comparison of resting-state networks. *Frontiers in systems neuroscience* **5**, 2 (2011).
3. Smith SM*, et al.* Network modelling methods for FMRI. *Neuroimage* **54**, 875-891 (2011).
4. van Erp TG*, et al.* Neuropsychological profile in adult schizophrenia measured with the CMINDS. *Psychiatry research* **230**, 826-834 (2015).
